# Experimental assessment of robust reference genes for qRT-PCR in lung cancer studies

**DOI:** 10.3389/fonc.2023.1178629

**Published:** 2023-05-18

**Authors:** Wei Gu, Yubin Wang, Ran Xu, Jiamin Li, Jingjie Jin, Jing Zhao, Yang Chen, Yuanzhi Lu, Gong Zhang

**Affiliations:** ^1^ Department of Pathology, First Affiliated Hospital of Jinan University, Jinan University, Guangzhou, China; ^2^ Key Laboratory of Functional Protein Research of Guangdong Higher Education Institutes and MOE Key Laboratory of Tumor Molecular Biology, Institute of Life and Health Engineering, Jinan University, Guangzhou, China

**Keywords:** qRT-PCR, reference gene, lung cancer, hypoxia, serum deprivation

## Abstract

Stable internal reference genes are crucial for quantitative real-time PCR (qRT-PCR) analyses in lung cancer studies. Widely used reference genes are mostly chosen by intuition or from pan-cancer transcriptome data and lack experimental validation by qRT-PCR in the context of lung cancer. This study evaluated the stability of candidate reference genes in lung cancer cell lines under normal homeostasis, hypoxia, and serum deprivation to screen for robust reference genes for qRT-PCR in lung cancer studies. The stability of reference gene combinations was also assessed. We found that most of the stably expressed genes from pan-cancer transcriptome analyses were not sufficiently stable under some of the tested conditions. *CIAO1*, *CNOT4*, and *SNW1* were found to be the most stable reference genes under various conditions. Greater stability was achieved by combining more reference genes. We further used the hypoxia biomarker hypoxia-inducible factor (HIF)-2α to demonstrate that choosing inappropriate reference genes can lead to incorrect qRT-PCR results. We also found that the stable reference genes were irrelevant to malignancy, which may explain their stability under various conditions that cancer cells often encounter. This study provides a list of validated and stable qRT-PCR reference genes and reference gene combinations for lung cancer that may standardize qRT-PCR experiments in future lung cancer studies.

## 1 Introduction

Lung cancer has the highest incidence rate worldwide and is among the most intensively studied cancers (1). Quantitative real-time PCR (qRT-PCR) is an important tool for quantifying gene expression levels in cancer cells and tissues and is essential in mechanistic studies, clinical diagnoses, and therapeutic target discovery (2). To obtain accurate quantitation, a proper reference gene for normalization, which is ideally stable under various physiological and pathological conditions, is crucial (3). However, the stability of reference genes has not been validated in most lung cancer studies. In this study, frequently used reference genes without any evidence of stability under specific experimental conditions were selected from the literature. In most lung cancer studies, reference genes that are frequently used in qRT-PCR are selected from the literature, and housekeeping genes are often used as reference genes because they are assumed to be expressed at a constant level under various conditions (4). However, commonly used housekeeping genes, such as *GAPDH* and β-actin (*ACTB*), have been evidenced to fluctuate across various tissues and under hypoxia and nutrient deprivation. The expression levels of *GAPDH* can vary by 80-fold between paired cancer and normal tissue samples in non-small cell lung cancer (5). *ACTB* dysregulation is commonly observed in malignancies, and the expression of the gene is upregulated in most cancer types (6). Using housekeeping genes as reference genes may therefore lead to misleading conclusions (7).

Recently, microarray and RNA sequencing (RNA-seq) datasets have been used to identify stable reference genes (8). As expected, transcriptomic pan-cancer datasets have presented novel candidate reference genes that have not been recognized in most cancer studies (9). However, different analyses have recommended different reference gene lists, even when using the same data source (such as The Cancer Genome Atlas [TCGA]), illustrating that the algorithm and mathematical models greatly influence the results. Moreover, most reference genes have not been experimentally validated in real-world studies. Owing to technological differences, the differentially expressed genes identified by transcriptomic methods have not been fully validated by qRT-PCR. To date, there is no practical guide for reference gene selection in lung cancer studies. Therefore, the experimental assessment of these reference genes is necessary.

Hypoxia and nutrient deprivation are typical microenvironmental features and universal phenomena in nearly all solid tumors. They can occur due to poor and/or competing blood supplies and are correlated with a more aggressive tumor phenotype and protection against oxidative stress (10, 11). Tumor cells under low-oxygen and serum-deprived conditions have been shown to display distinct features in biomarker profiles and signaling networks and sensitivity to targeted drugs compared to those under ambient air and are more similar to *in vivo* tissues and tumors (12). The actual oxygen content of physoxia is approximately 5%; pathological hypoxia in tumors is typically below 2% and often <0.1% for tumor cells. Serum deprivation has been shown to correspond to the presence of 0.5% fetal bovine serum (FBS) or no FBS (10, 13, 14). However, most *in vitro* studies on cancer cell models have been performed with ambient atmospheric oxygen (~21%) and 10% FBS. Gene expression under low-oxygen or serum-deprived conditions changes compared to that under normal conditions, and several traditional reference genes have been found to be unstable under these conditions. Under hypoxic conditions, *GAPDH* and *ACTB* mRNA expression has been found to increase by 21.2%–75.1% and 5.6%–27.3%, respectively (15). Therefore, it is necessary to validate qRT-PCR reference genes in lung cell lines under hypoxic and serum-deprived conditions to improve the method for future studies.

In this study, we performed qRT-PCR experiments on lung cancer cell lines (A549, H1299, H358, H441, and H460) and normal lung cell lines (Beas-2B, HBE, and HULEC-5a) under normal homeostasis, hypoxia, and serum-deprived conditions to screen for robust reference genes for qRT-PCR in lung cancer studies. These cell lines and conditions are typical for lung cancer studies. This study validated novel and robust qRT-PCR reference genes and reference gene combinations for lung cancer research under various culture conditions and is therefore valuable for gene expression studies and functional lung cancer research.

## 2 Materials and methods

### 2.1 Selection of candidate reference genes

A total of 10 candidate reference genes were selected from the top 10 most stably expressed reference genes (*RBM45*, *NRF1*, *BRAP*, *WDR33*, *CNOT2*, *TIAL1*, *CIAO1*, *TARDBP*, *ZNF207*, and *HNRNPK*) from previous pan-cancer studies, including TCGA and Cancer Cell Line Encyclopedia RNA-seq data (16). The singscore R package was installed in Bioconductor, and the getStableGenes(10) function was used to obtain the top 10 most stably expressed reference genes. We included five stable reference genes (*UBC*, *ACTB*, *PUM1*, *RPN1*, and *RPL13A*) in lung adenocarcinoma (LUAD) from another pan-cancer reference gene study that focused on TCGA RNA-seq data (9). Five recommended stable reference genes (*PUM1*, *IPO8*, *HNRNPL*, *SNW1*, and *CNOT4*) were included in the human cancer and normal cell lines (17). We also included the top 3 most stable reference genes (*ACTB*, *PPIA*, and *PGK1*) from a previous study on lung cancer (4). Related information on the candidate reference genes is shown in **Table 1**.

**Table 1:** Candidate genes, primer sequences, and PCR efficiency.

Symbol	Ensembl gene ID	Forward primer	Reverse primer	Amplicon size (bp)	Exon location	PCR efficiency (%)
*RBM45*	ENSG00000155636	CCAAGGAGTCCAAGGGCATT	CCAGATGATCGGGACTGAGC	145	Intron-flank, 1st, 2nd	100
*NRF1*	ENSG00000106459	GGTCGCAGTCTCCACGG	TTTGGGTCACTCCGTGTTCC	79	Intron-flank, 1st, 2nd	105
*BRAP*	ENSG00000089234	CGGCCGGGGAAATGTCT	TACTTCTTTGCGCAGTGGGG	231	Intron-span, 1st, 3rd	108
*WDR33*	ENSG00000136709	TGGGGAGTTTACCCTGTGGA	ACATATCCTCCGTGGTCTGC	135	Intron-flank, 5th, 6th	106
*CNOT2*	ENSG00000111596	ACTACCAGGTGACAAACAGC	TAGTCACTGTCGACCCCCTC	70	Intron-flank, 3rd, 4th	107
*TIAL1*	ENSG00000151923	CCCTGAGGTGGACCACATTT	AAGCGACTCCAAAATCACGC	78	Exonic, 13th	107
*CIAO1*	ENSG00000144021	TTGGGTCTGGGAAGTTGATGA	CTCAAGGGTGGCACAGCATA	187	Intron-span, 3rd, 6th	95
*TARDBP*	ENSG00000120948	TCGGGGACCTCCAAAGACTA	AACCATGCCGTTGACAAGTT	75	Exonic, 6th	105
*ZNF207*	ENSG00000010244	CAAAGTGGATGCCAACTGCC	CCAAAGTTGCCAGCCCTACT	117	Exonic, 11th	98
*HNRNPK*	ENSG00000165119	GTCTCGCGCGGCTACTG	CCCCAGCATTCTTGCTCTGA	283	Intron-span, 1th, 5th	97
UBC*	ENSG00000150991	ACGGGACTTGGGTGACTCTA	ATCGCCGAGAAGGGACTACT	82	Exonic, 1st	108
ACTB*,**,#	ENSG00000075624	GAAGATCAAGATCATTGCTCCT	TACTCCTGCTTGCTGATCCA	111	Intron-span, 5th, 6th	91.8
PUM1*,***,##	ENSG00000134644	TGCGGGAGATTGCTGGACAT	GTGTGGCACGCTCCAGTTTC	87	Intron-flank, 15th, 16th	98.4
RPN1*	ENSG00000163902	CTGACTGTGAAGATCATCCTGCC	GTCCAGATAGGTGTAGTGCAGC	105	Intron-flank, 6th, 7th	102
RPL13A*,#	ENSG00000142541	CGAGGTTGGCTGGAAGTACC	CTTCTCGGCCTGTTTCCGTAG	121	Intron-flank, 6th, 7th	103
PPIA**,#	ENSG00000196262	TCCTGGCATCTTGTCCAT	TGCTGGTCTTGCCATTCCT	179	Intron-flank, 5th, 6th	107
PGK1**,#	ENSG00000102144	GCCACTTGCTGTGCCAAATG	CCCAGGAAGGACTTTACCTT	102	Intron-span, 10th, 11th	139
IPO8***,##	ENSG00000133704	GGCATACAGTTTAACCTGCCAC	CAGGAGAGGCATCATGTCTGTAA	118	Intron-flank, 14th, 15th	92.5
HNRNPL***,##	ENSG00000104824	CCAAGGCCTCTCTCAATGGG	TTCAAGCGTGTAGGCTTTGC	82	Intron-span, 5th, 6th	97.9
SNW1***,##	ENSG00000100603	GCAGCTCCTGATAAGAGGTCG	CCGAGGATTAGGAACACCGAG	87	Intron-span, 11th, 12th	95.8
CNOT4***,##	ENSG00000080802	GTCCAAAACCTGACTGCATGTATC	GGTGTTTACCCGCCTGCAT	87	Intron-span, 7th, 8th	96.3

One to 10 candidate genes from reference (16).

*Five recommended stable reference genes for LUAD from reference (Krasnov, Kudryavtseva et al., 2019).

**Three recommended stable reference genes for lung cancer from reference (Ali, Du et al., 2015).

***Five recommended stable reference genes for human cancer and normal cell lines from reference (Racz, Nagy et al., 2021).

^#^Primers from reference (Ali, Du et al., 2015).

^##^Primers from reference (Racz, Nagy et al., 2021).

UBC primers from reference (Augustyniak, Lenart et al., 2019) and RPN1 primers from OriGen.

### 2.2 Cell lines and treatment

The normal human lung cell lines, BEAS-2B and HBE, were purchased from the American Type Culture Collection (ATCC). The human lung cancer cell lines A549, NCI-H1299, NCI-H358, NCI-H441, and NCI-H460 were purchased from the ATCC. The normal human lung cell line, Hulec-5a, was purchased from Meisen CTCC (Zhejiang Meisen Cell Technology Co., Ltd.). These cell lines are frequently used in lung cancer research.

Hulec-5a cells were cultured in specific medium (Procell), which is based on MCDB13 medium, supplemented with 10% FBS, 10 ng/ml epidermal growth factor, 1 g/ml hydrocortisone, 10 mM L-glutamine, and 1% penicillin/streptomycin (Gibco-BRL). The other cells were cultured in Dulbecco’s modified Eagle’s medium (Gibco-BRL, Carlsbad, CA, USA) supplemented with 10% FBS (Gibco-BRL) and 1% penicillin/streptomycin (Gibco-BRL).

Hypoxic culture conditions were created using AnaeroPack (MITSUBISHI, Japan) according to the manufacturer’s instructions. The normoxic culture comprised 20.9% O_2_ and 5% CO_2_. Physoxic (~5% O_2_) and hypoxic (<1% O_2_) culture conditions were created using AnaeroPack and cultured for 24 h. Serum deprivation was performed using 0.5% and 0% serum.

The cultured cells were routinely tested for mycoplasma contamination. The cell line identities were confirmed using short tandem repeat (STR) profiling.

### 2.3 RNA extraction and cDNA synthesis

Total RNA was extracted using TRIzol reagent (Waltham, Massachusetts, USA, Invitrogen) with 5 × 10^6^ cells from each cell line according to the manufacturer’s instructions. The quality and quantity of RNA were determined using a NanoDrop (Waltham, Massachusetts, USA, Thermo Scientific) at OD260/280 and OD260/230 ratios. Integrity and genomic DNA contamination were assessed using agarose gel electrophoresis (**Supplementary Figure S1**). cDNA synthesis was performed using the HiScript^®^ III RT SuperMix (Nanjing, China, Vazyme) according to the manufacturer’s instructions, which contained the genomic DNA degradation procedure.

### 2.4 Design and evaluation of primers

Primer sequences were designed using the NCBI primer design tool. The specificities of the primers were confirmed using NCBI Primer-Blast. The RT-PCR products were subjected to 2% agarose gel electrophoresis and a melting curve analysis to evaluate their lengths and specificities. The amplification efficiency was evaluated using a standard curve analysis of fivefold serial dilutions. The specific primers that were closest to an amplification efficiency of 100% were selected for further analysis (**Table 1**).

### 2.5 Quantitative real-time PCR

qRT-PCR was performed with the ChamQ Universal SYBR qPCR Master Mix (Vazyme) in 96-well reaction plates and Bio-Rad Real-Time PCR System according to the manufacturer’s instructions. The gene expression profiles were assessed using the –ΔΔCt method. qRT-PCR was performed using three biological and three technical replicates.

### 2.6 PCR efficiency determination

A series of five-point 10-fold dilutions of cDNA were prepared and introduced into the qRT-PCR. The Cq values were plotted against the logarithm of the base 10 concentration, and the slopes of the curves and regression coefficients were determined. The PCR efficiency values were calculated using the formula E(%) = [10^(1/−slope)−1^] × 100%.

### 2.7 Stability evaluation and statistical analysis of qRT-PCR results

RefFinder (18), geNorm (19), NormFinder (20), BestKeeper (21), and Comprehensive delta Ct (22) were used to analyze the stability of the reference genes.

### 2.8 Protein–protein interaction analysis

A protein–protein interaction (PPI) analysis was performed using the Search Tool for the Retrieval of Interacting Genes (STRING) (https://string-db.org) database (23). The nodes in the networks corresponded to the proteins, and the edges represented the PPIs.

## 3 Results

### 3.1 Selection of candidate reference genes and primer evaluation

Candidate reference genes were selected from previous studies aimed at identifying stably expressed genes using pan-cancer transcriptome data (9, 16). The top 10 most stable reference genes (*RBM45*, *NRF1*, *BRAP*, *WDR33*, *CNOT2*, *TIAL1*, *CIAO1*, *TARDBP*, *ZNF207*, and *HNRNPK*) were selected from the singscore R package developed by Bhuva et al. (16). Five recommended stable reference genes for LUAD (*UBC*, *ACTB*, *PUM1*, *RPN1*, and *RPL13A*) were selected (9). Validated stable qRT-PCR reference genes in lung cancer were included in the stability evaluation (4, 17). Primers for qRT-PCR were obtained from the literature, if available; otherwise, we designed the primers (**Table 1**). We used 1 μg of high-quality total RNA from each cell line as the input. DNase-treated mRNA was reverse-transcribed into cDNA using oligo-dT and random primers. All of the Cq values ranged from 15 to 30 (**Supplementary Figure S2**), which is within the linear range of most instruments. Primer specificity was confirmed using agarose gel electrophoresis (**Supplementary Figure S3**) and a melting curve analysis (**Supplementary Figure S5**) after the PCR. The amplification efficiency of each primer pair was measured using serial dilutions of cDNA and RT-qPCR (**Table 1**). Apart from that of PGK1, the amplification efficiencies of the genes ranged from 91.8% to 108% and were mostly within the range of 95%–105%, which ensured accurate quantification.

### 3.2 Expression stability of the candidate reference genes under various conditions

**Figure 1:**
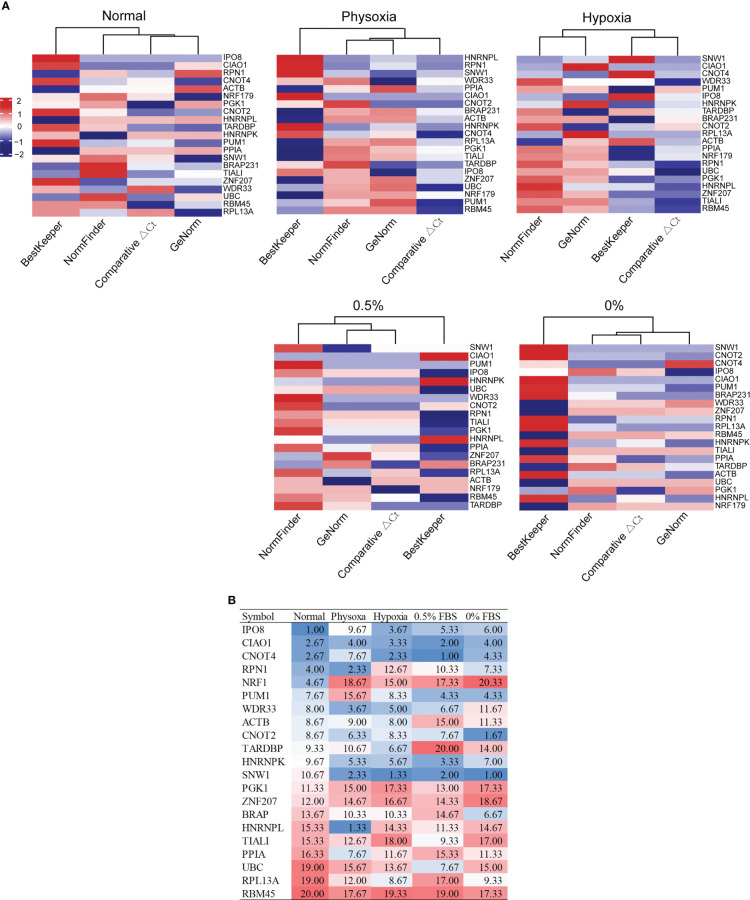
Expression stability of candidate reference genes under various conditions. **(A)** Ranks of the candidate reference genes under five conditions, calculated using four algorithms. **(B)** Average ranks of three algorithms (comparative ΔCt, geNorm, and NormFinder) under five conditions.

We performed qRT-PCR for the 21 candidate reference genes in three normal lung cell lines and five lung cancer cell lines under various culture conditions, including normal, physoxic (~5%), pathological hypoxic (<1% O_2_), and serum deprivation (0.5% and 0% FBS) conditions, starting with 1 μg of total RNA. All of the Cq values were within the range of 15–30, which was suitable for quantification using qRT-PCR (**Supplementary Figure S2**). For each condition, we used four methods (geNorm, NormFinder, ΔCt, and BestKeeper) and a web-based reference to evaluate gene expression stability and determine the rank of each candidate reference gene under each condition (**Supplementary Table S2**). The ranks calculated using the BestKeeper algorithm deviated markedly from those calculated using the other three algorithms (**Figure 1A**). Therefore, we excluded BestKeeper from subsequent analyses. We then calculated the average ranks of the three algorithms (geNorm, NormFinder, and Ct) for each candidate reference gene under each condition (**Figure 1B**).

Under the normal condition, the five most stable reference genes were *IPO8*, *CIAO1*, *CNOT4*, *RPN1*, and *NRF1*. The stable reference genes that ranked 6–10 were *PUM1*, *WDR33*, *ACTB*, *CNOT2*, and *TARDBP*. Under the physoxic condition, the top 10 most stable reference genes were *HNRNPL*, *RPN1*, *SNW1*, *WDR33*, *CIAO1*, *HNRNPK*, *CNOT2*, *PPIA*, *CNOT4*, and *ACTB*. Under the hypoxic condition, the top 10 most stable reference genes were *SNW1*, *CNOT4*, *CIAO1*, *IPO8*, *WDR33*, *HNRNPK*, *TARDBP*, *ACTB*, *PUM1*, and *CNOT2*. Under the 0.5% FBS condition, the top 10 most stable reference genes were *CNOT4*, *SNW1*, *CIAO1*, *HNRNPK*, *PUM1*, *IPO8*, *WDR33*, *UBC*, *CNOT2*, and *TIAL1*. Under the 0% FBS condition, the top 10 most stable reference genes were *SNW1*, *CNOT2*, *CIAO1*, *CNOT4*, *PUM1*, *IPO8*, *BRAP*, *HNRNPK*, *RPN1*, and *RPL13A*.

Although almost all of the genes were stable under some of the conditions and not as stable under the others, several of the reference genes ranked relatively high under various conditions. Only *CIAO1* ranked in the top 5 under the five different conditions; therefore, *CIAO1* was considered the most stable reference gene under the five conditions. *CIAO1* is a novel candidate reference gene that was identified from pan-cancer transcriptome data (16). *CNOT4* and *CNOT2* had average ranks of <10 under all of the conditions and may therefore be good universal reference genes. *CNOT2* is also a novel candidate reference gene that was identified from pan-cancer transcriptome data (16). *CNOT4* ranked in the top 5 most stable genes under the four conditions and only ranked ninth under physoxia. Therefore, *CNOT4* and *CNOT2* may be stable reference genes for lung cancer cells under these five conditions. *SNW1* was also stable under all of the stress conditions (average rank < 2.5) but ranked 10.67 under the normal condition; thus, it is also a good candidate.

### 3.3 Unstable reference genes may lead to misleading conclusions for hypoxia molecular biomarkers

Hypoxia-inducible factor (HIF) is a dimer transcription factor that mediates adaptive responses to changes in oxygen levels and is composed of an oxygen-regulated subunit and a constitutively expressed subunit (24). HIF-2α is one of three isoforms of the HIF-α subunit and can form a complex with HIF-1β. HIF-2α is specifically expressed in certain tissues, including the lungs (25). Therefore, HIF-2α was chosen as a hypoxia molecular biomarker to test the stability of the reference genes.

**Figure 2:**
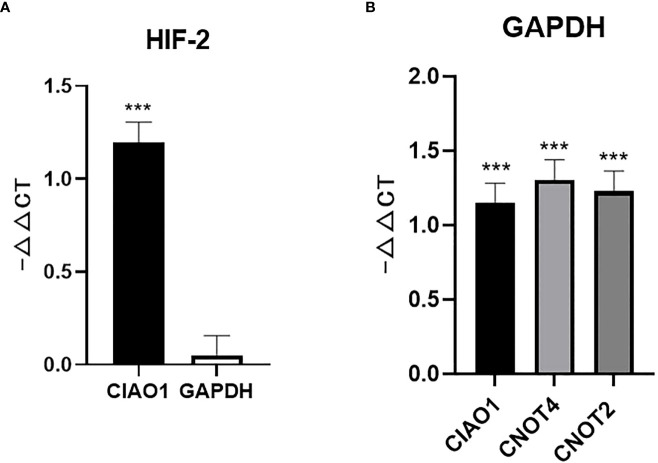
Unstable reference genes may lead to misleading conclusions for molecular biomarker expression. **(A)** HIF-2α expression level determined using *CIAO1* and *GAPDH* as reference genes. **(B)** *GAPDH* expression determined using *CIAO1*, CIAO4, and *CNOT2* as reference genes. *** indicate statistical significance (p<0.001).

When comparing the expression of the genes under physoxia (5% O_2_) and the normal condition, the expression of HIF-2α exhibited no significant difference when using the widely used *GAPDH* as reference, which contradicted previously published results (25). When using the stable reference gene *CIAO1*, the expression of HIF-2α significantly increased under physoxia, which coincided with previously reported conclusions (**Figure 2A**). *CIAO1*, *CNOT2*, and *RBM45* were the candidate reference genes used in this study and were screened as stably expressed reference genes using pan-cancer transcriptome data (16). The failure of using *GAPDH* was due to its expression being significantly increased under the physoxic condition, which was confirmed by qRT-PCR using the reference genes *CIAO1*, *CNOT4*, and *CNOT2* (**Figure 2B**). These three reference genes were ranked among the top 10 most stable genes under both the physoxic and hypoxic conditions. These results suggest that the traditional reference gene *GAPDH* should not be used as a reference gene in hypoxia-related research, whereas the stable reference genes confirmed in this study under low-oxygen conditions are recommended.

### 3.4 Reference gene combinations exhibit better stability

**Figure 3:**
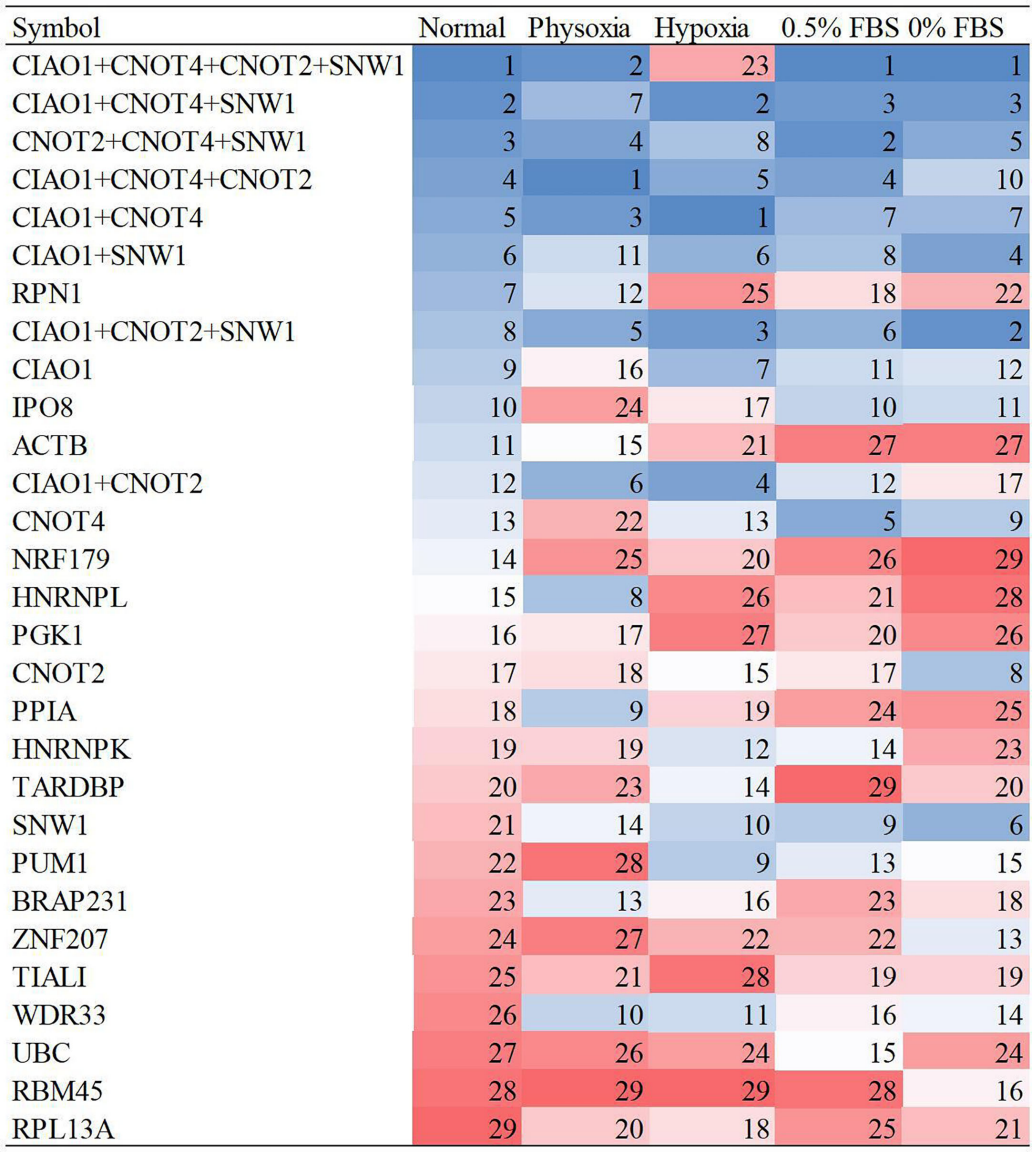
Stability ranks of reference gene combinations under five conditions.

In qRT-PCR, reference gene combinations are more stable than single reference genes because of their lower susceptibility to perturbation (26). Therefore, it is necessary to evaluate the stability of reference gene combinations compared to that of single reference genes. Pairwise variations in geNorm indicated the optimal number of reference genes (27). The analysis revealed that the combination of four reference genes was optimal when comprehensively considering the five conditions (**Supplementary Table S3**). Fewer reference genes are more convenient for experimentation. Therefore, three reference gene combinations and two reference gene combinations were included in the stability evaluation. The top 4 reference genes (*CIAO1*, *CNOT2*, *CNOT4*, and *SNW1*) were grouped into four, three, and two reference gene combinations. The average stability ranks, excluding that determined by BestKeeper, are shown in **Figure 3**. The results revealed that the four reference gene combinations were the most stable, followed by the three reference gene combinations, and then the two reference gene combinations.

The four-gene combination (*CIAO1*+*CNOT2*+*CNOT4*+*SNW1*) showed the highest stability under the five culture conditions; therefore, this combination is highly recommended for qRT-PCR. The three- and two-gene combinations also exhibited better stability than the single reference genes and are recommended for qRT-PCR because they are less complex than the four-gene combination. The recommended three-gene combination includes *CIAO1*, *CNOT4*, and *SNW1*, and the recommended two-gene combination includes *CIAO1* and *CNOT4*.

### 3.5 Stable reference genes are irrelevant to cancer malignancy

**Figure 4:**
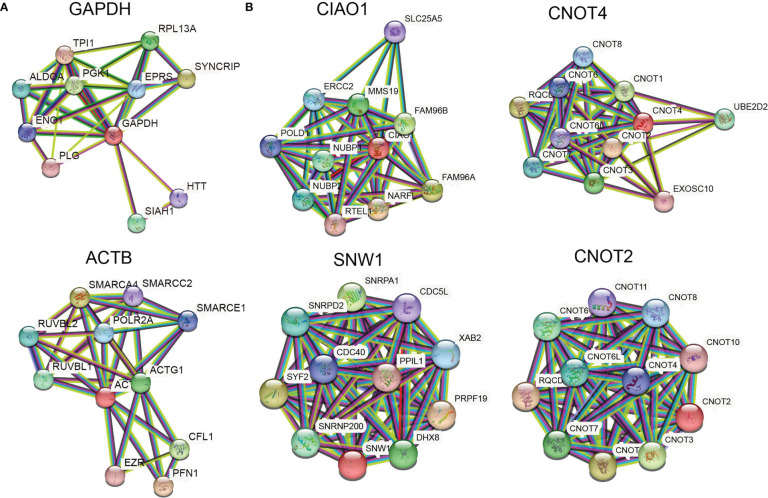
Stable reference genes are irrelevant to malignancy. **(A)** PPI networks of *GAPDH* and *ACTB*. **(B)** PPI networks of *CIAO1*, *CNOT4*, *SNW1*, and *CNOT2*.

Ideally, reference genes should be irrelevant to major phenotype-relevant functions in investigated cells. *GAPDH* is a key gene in central carbon metabolism and energy pathways, especially the glycolysis pathway. It is well known that cancer cells tend to shift their energy production from aerobic respiration to glycolysis (the Warburg effect) and that *GAPDH* expression is upregulated in cancer cells, especially under physoxic conditions. Therefore, *GAPDH* is not considered a stable gene in cancer. Similarly, when cell skeletons change, *ACTB* also changes. The PPI networks of *GAPDH* and *ACTB* showed less significant PPI enrichment (*p* > 0.001) and clear KEGG pathway enrichment (**Figure 4A**; **Table 2**). The enrichment of the entire *ACTB* KEGG pathway is shown in **Supplementary Figure S7**. In contrast, the stable reference genes validated in this study (*CIAO1*, *CNOT4*, *SNW1*, and *CNOT2*) exhibited tight PPI interactions in the clusters (PPI enrichment, *p* < 1e–16) and no significant KEGG pathway enrichment, suggesting that these clusters are irrelevant to malignancy (**Figure 4B**; **Supplementary Figure S8**). This may explain the stability of these genes in cancer cells.

**Table 2:** KEGG pathway enrichment analysis of the *GAPDH* and *ACTB* PPI networks.

Reference gene	Pathway	Description	Count in network	Strength	False discovery rate
GAPDH	hsa00010	Glycolysis/gluconeogenesis	5 of 65	2.14	7.71E−08
	hsa01230	Biosynthesis of amino acids	5 of 73	2.09	7.71E−08
	hsa00051	Fructose and mannose metabolism	2 of 32	2.05	8.90E−03
	hsa01200	Carbon metabolism	5 of 117	1.88	4.35E−07
	hsa04066	HIF-1 signaling pathway	4 of 106	1.83	2.54E−05
	hsa01100	Metabolic pathways	6 of 1447	0.87	3.70E−03
ACTB	hsa05110	Vibrio cholerae infection	2 of 48	1.87	9.70E−03
	hsa04971	Gastric acid secretion	3 of 73	1.86	7.60E−04
	hsa05416	Viral myocarditis	2 of 55	1.81	1.16E−02
	hsa05225	Hepatocellular carcinoma	5 of 160	1.74	5.96E−06
	hsa04520	Adherens junction	2 of 67	1.73	1.58E−02
	hsa05100	Bacterial invasion of epithelial cells	2 of 70	1.71	1.61E−02
	hsa04670	Leukocyte transendothelial migration	3 of 109	1.69	2.00E−03
	hsa05412	Arrhythmogenic right ventricular cardiomyopathy	2 of 76	1.67	1.77E−02
	hsa04810	Regulation of actin cytoskeleton	5 of 209	1.63	1.10E−05
	hsa05410	Hypertrophic cardiomyopathy	2 of 89	1.6	2.13E−02
	hsa04714	Thermogenesis	5 of 229	1.59	1.14E−05
	hsa05414	Dilated cardiomyopathy	2 of 95	1.57	2.30E−02
	hsa04530	Tight junction	3 of 156	1.53	4.60E−03

## 4 Discussion

Although next-generation sequencing (NGS) has been widely commercialized and its cost has decreased, its complexity, accuracy, sensitivity, speed, and cost still cannot match those of conventional qRT-PCR for the quantification of a few genes. Although many studies have warned that the widely used *GAPDH* is not stable and should not be used as a reference gene in cancer research, thousands of papers each year still use it without validation. Various studies based on NGS data have recommended several alternative reference genes; however, these genes have not been experimentally evaluated or validated using qRT-PCR. Indeed, the validation of a reference gene is not simple. In many cases, this process can be more complicated than the study itself. Moreover, pan-cancer calculations tend to be biased by the system bias of multicenter NGS data. Complex algorithms can effectively correct such system bias (28), but this necessitates raw NGS data, which are not publicly available, such as in TCGA, due to legal issues. This hinders the widespread application of stable reference genes. Cancer research using qRT-PCR is mostly function oriented and thus unlikely to involve pan-cancer analyses. Therefore, cancer-type-specific reference genes are more practical for research.

In this study, stably expressed candidate reference genes suggested by recent pan-cancer investigations were validated by qRT-PCR in lung cancer cells under five different culture conditions. Stable qRT-PCR reference genes and gene combinations were identified and included 21 candidates, including novel candidates from pan-cancer transcriptome data and validated qRT-PCR reference genes reported in previous lung cancer studies. *CIAO1*, *CNOT4*, *SNW1*, and *CNOT2* were the most stably expressed reference genes under the various conditions. *CIAO1* and *CNOT2* were novel candidates that have not been previously reported.

The stable qRT-PCR reference genes validated in this study are suitable for normalizing and quantifying gene expression levels in lung cancer research involving hypoxic and serum-deprived conditions. Hypoxia and serum deprivation are conditions that are more relevant to physical tumor tissues. The experimental results obtained under hypoxia and serum deprivation resembled those from real clinical situations and necessitated accurate normalization. The stably expressed reference genes validated in this study under hypoxic and serum-deprived conditions are valuable for more realistic lung cancer studies.

*CIAO1* assists in the incorporation of iron–sulfur clusters into cytoplasmic and nuclear iron–sulfur proteins. *CNOT4* and *CNOT2* are subunits of the CCR4–NOT complex. The CCR4–NOT complex is a global transcriptional regulator that participates in translational monitoring and mRNA degradation (29). *CNOT4* exhibits E3 ubiquitin ligase activity and ubiquitinates ribosomal subunit proteins. *CNOT2* interacts with histone deacetylases and represses the transcription of polymerase II. *SNW1* not only functions as a coactivator that enhances the transcription of some polymerase II promoters but also functions as a splicing factor that controls specific gene expression. These four reference genes are essential for cell life activities and irrelevant to malignancy, which may explain why these genes exhibited greater stability than the other candidates. These results suggest a general rule for selecting reference genes in other scenarios.

In summary, we validated stably expressed reference genes and reference gene combinations for use in lung cancer research involving various conditions. The reference genes and combinations recommended in this study may standardize future studies on lung cancer gene expression.

## Data availability statement

The original contributions presented in the study are included in the article/**Supplementary Material**. Further inquiries can be directed to the corresponding authors.

## Author contributions

WG and YW contributed to the conception of the study. WG, YW, and RX designed the study. WG, YW, RX, and JL performed the experiments. WG, YW, and RX analyzed the data. JJ, JZ, YC, and YL supported this project. WG and GZ prepared the manuscript. All authors contributed to the revision of the manuscript and have read and approved the submitted version.
