# Supplementary material for: Experimental assessment of robust reference genes for qRT-PCR in lung cancer studies
Source: Front Oncol. 2023 May 18;13:1178629. doi: 10.3389/fonc.2023.1178629 (PMC10233025; doi:10.3389/fonc.2023.1178629)
Supplement: Supplementary file 1 [file DataSheet_1.pdf]

Supplementary Material

Experimental assessment of robust reference genes in qRT-PCR in lung cancer

Wei Gu[1,2†], Yubin Wang[2†], Ran Xu[2†], Jiamin Li[2], Jingjie Jin[2], Jing Zhao[2], Yang Chen[2], Yuanzhi Lu[1*], Gong Zhang[2*]

* **Correspondence:** Gong Zhang: zhanggong-uni@qq.com. Yuanzhi Lu: Yuanzhi.lu@jnu.edu.cn.

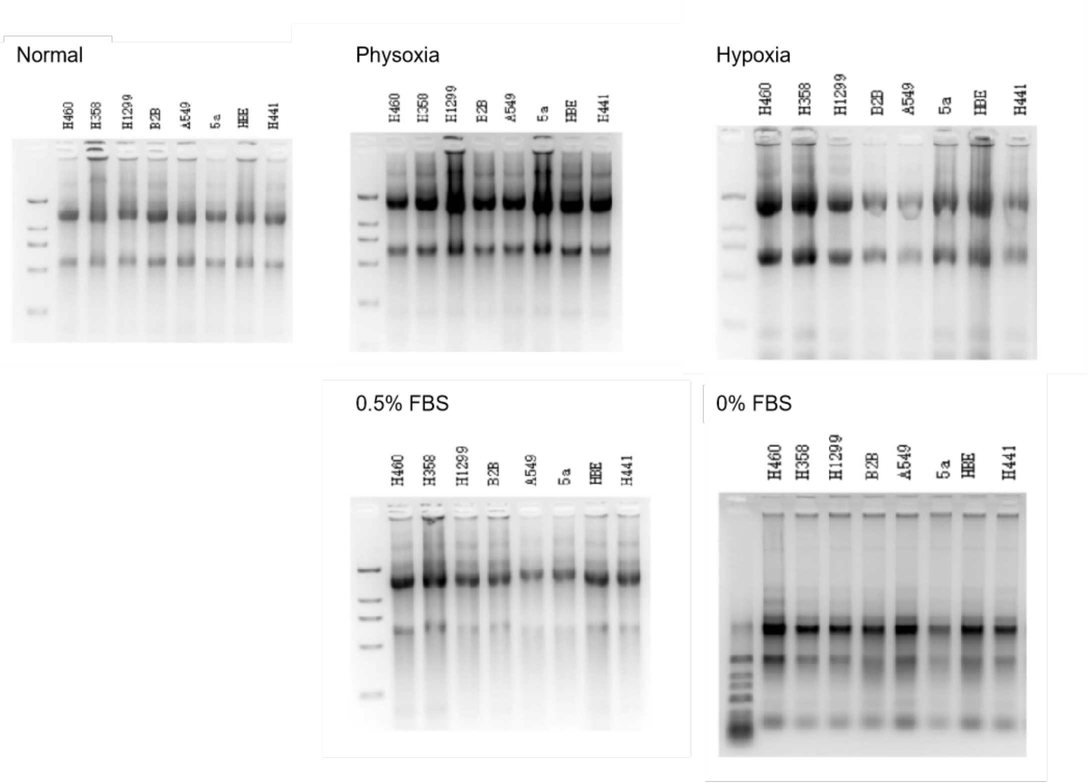

Supplementary Figure 1. Agrose electrophoresis image of isolated RNA samples.

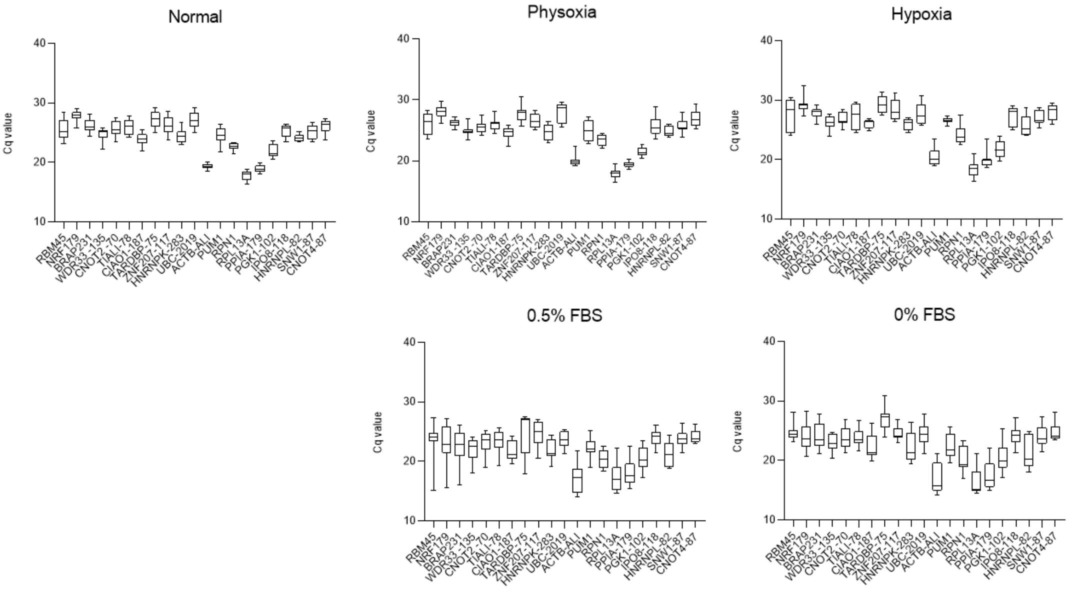

Supplementary Figure 2. Expression level of selected candidate reference under five conditions.

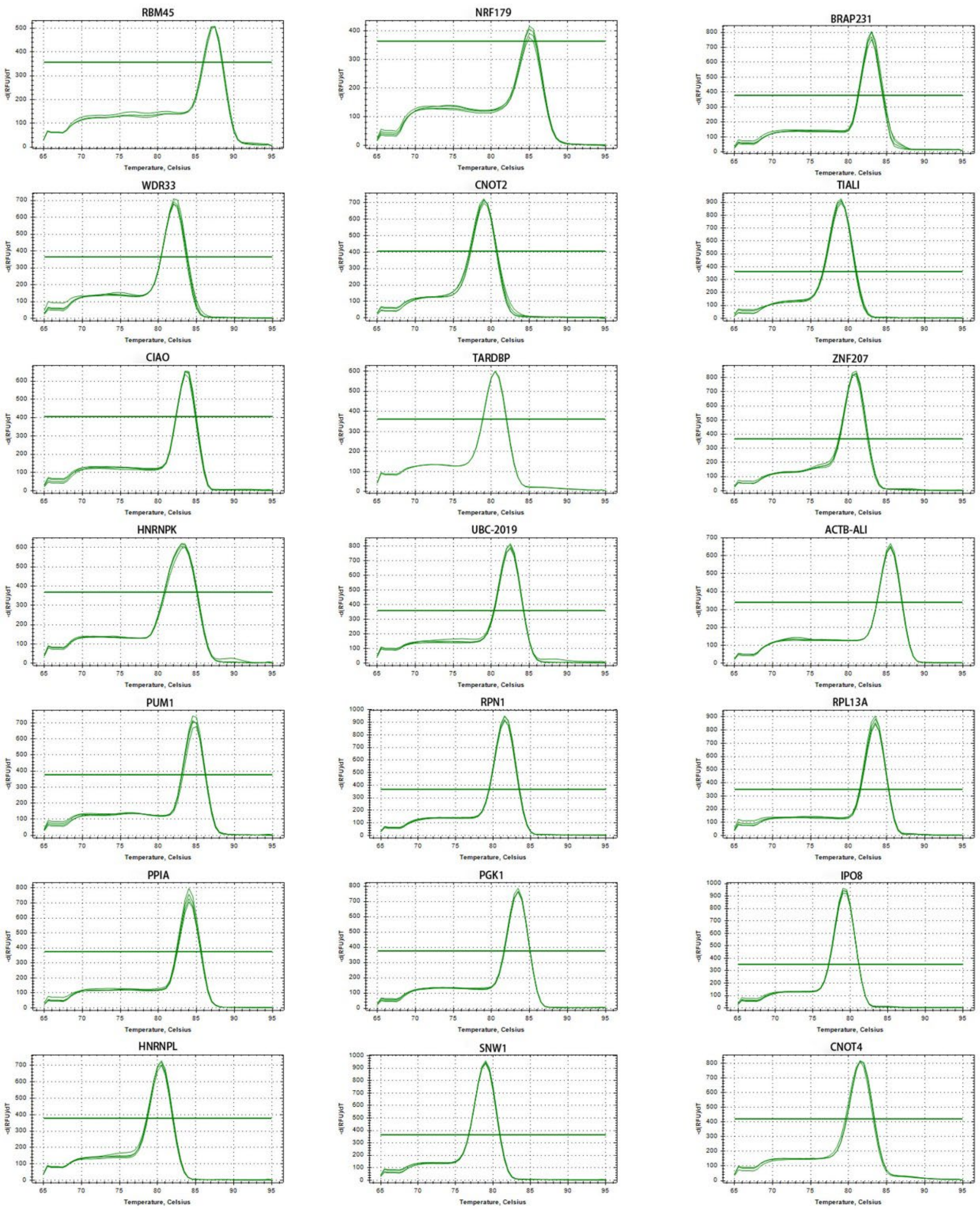

Supplementary Figure 3. Melting curves of candidate reference genes after amplification.

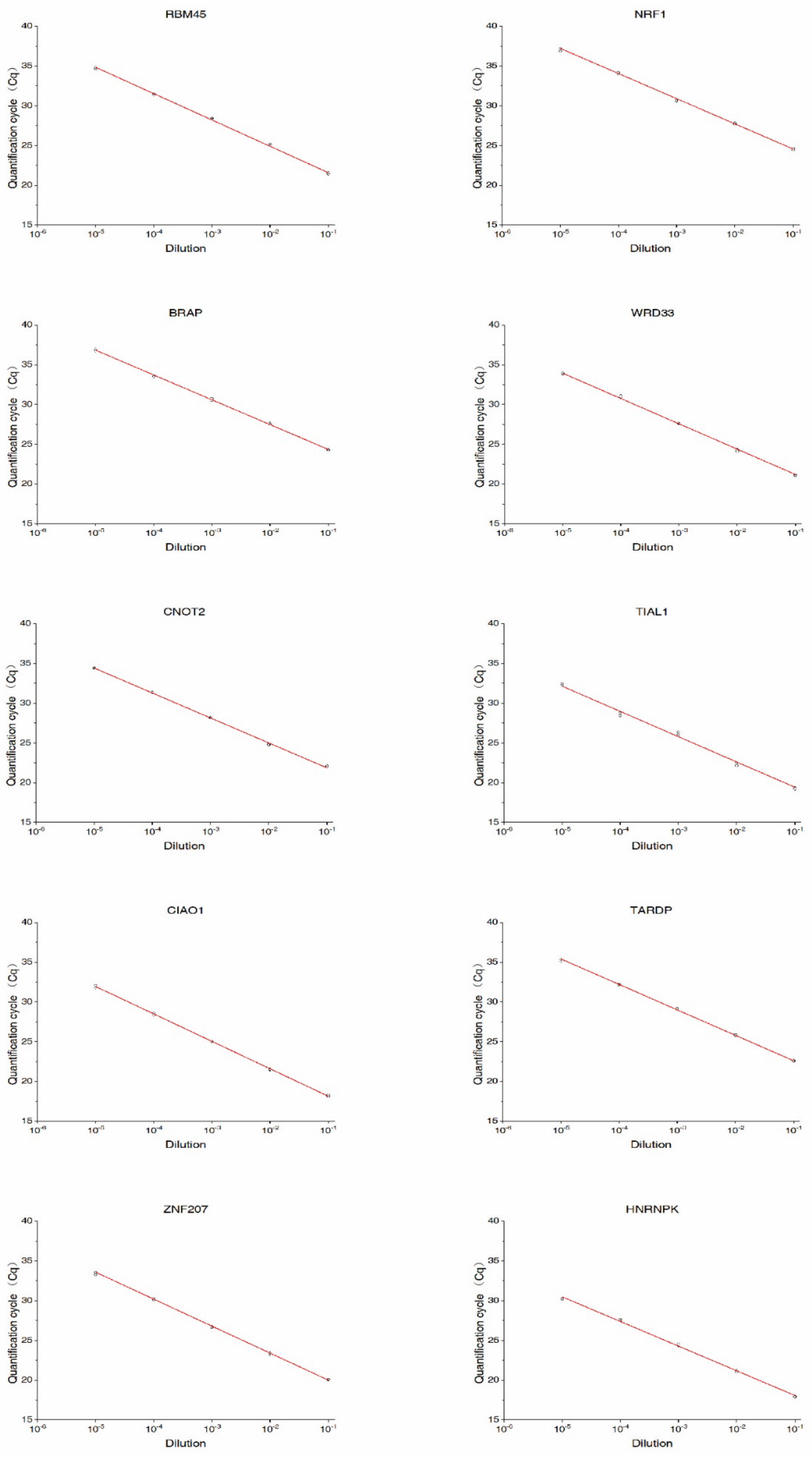

Supplementary Figure 4. Amplification efficiencies of 10 candidate reference gene primers which designed in this study.

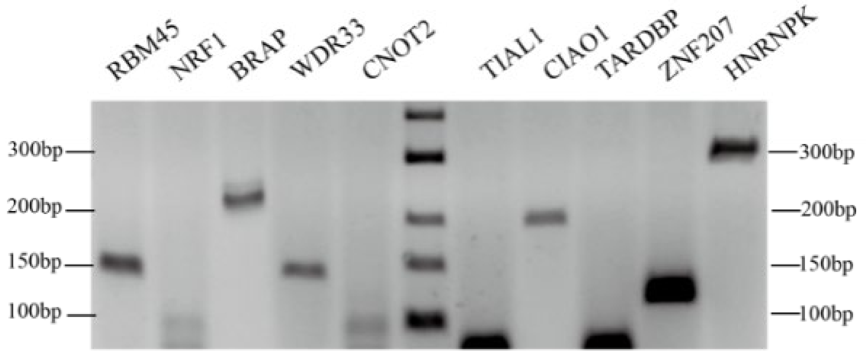

Supplementary Figure 5. Agrose electrophoresis image of PCR products amplificated by designed qPCR primers.

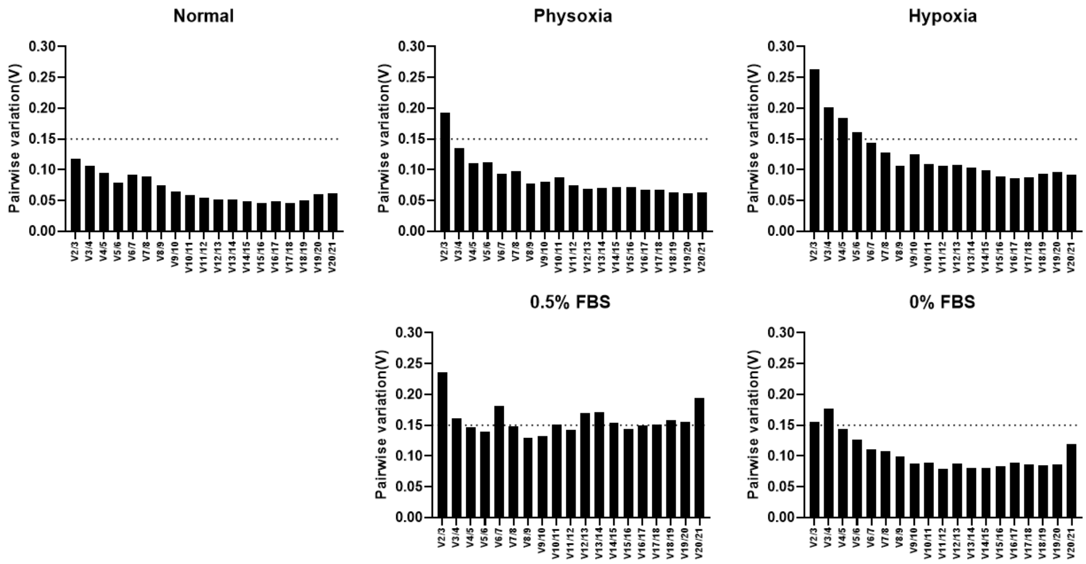

Supplementary Figure 6. Optimal number of reference genes for accurate normalization as determined by geNorm.

KEGG Pathways

pathway	description	count in network	strength	false discovery rate
hsa05110	Vibrio cholerae infection	2 of 48	1.87	0.0097
hsa04971	Gastric acid secretion	3 of 73	1.86	0.00076
hsa05416	Viral myocarditis	2 of 55	1.81	0.0116
hsa05225	Hepatocellular carcinoma	5 of 160	1.74	5.96e-06
hsa04520	Adherens junction	2 of 67	1.73	0.0158
hsa05100	Bacterial invasion of epithelial cells	2 of 70	1.71	0.0161
hsa04670	Leukocyte transendothelial migration	3 of 109	1.69	0.0020
hsa05412	Arrhythmogenic right ventricular cardiomyopathy	2 of 76	1.67	0.0177
hsa04810	Regulation of actin cytoskeleton	5 of 209	1.63	1.10e-05
hsa05410	Hypertrophic cardiomyopathy	2 of 89	1.6	0.0213
hsa04714	Thermogenesis	5 of 229	1.59	1.14e-05
hsa05414	Dilated cardiomyopathy	2 of 95	1.57	0.0230
hsa04530	Tight junction	3 of 156	1.53	0.0046
hsa04919	Thyroid hormone signaling pathway	2 of 119	1.48	0.0338
hsa05130	Pathogenic Escherichia coli infection	3 of 187	1.46	0.0067
hsa04611	Platelet activation	2 of 122	1.46	0.0338
hsa05135	Yersinia infection	2 of 125	1.45	0.0338
hsa05418	Fluid shear stress and atherosclerosis	2 of 130	1.44	0.0348
hsa05205	Proteoglycans in cancer	3 of 196	1.43	0.0068
hsa04210	Apoptosis	2 of 132	1.43	0.0348
hsa04015	Rap1 signaling pathway	3 of 202	1.42	0.0068
hsa05132	Salmonella infection	3 of 209	1.41	0.0068
hsa04145	Phagosome	2 of 142	1.4	0.0380
hsa05131	Shigellosis	3 of 218	1.39	0.0068
hsa04921	Oxytocin signaling pathway	2 of 149	1.38	0.0401
hsa04390	Hippo signaling pathway	2 of 153	1.37	0.0407
hsa05164	Influenza A	2 of 165	1.33	0.0454
hsa05014	Amyotrophic lateral sclerosis	3 of 352	1.18	0.0177

(less ...)

Supplementary Figure 7. *ACTB* KEGG pathway enrichment.

A

KEGG Pathways

pathway	description	count in network	strength	false discovery rate
hsa03018	RNA degradation	10 of 75	2.38	5.07e-21

B

KEGG Pathways

pathway	description	count in network	strength	false discovery rate
hsa03040	Spliceosome	11 of 132	2.17	7.17e-22

Supplementary Figure 8. KEGG pathway enrichment of stable reference genes. **(A)** *CNOT4* and *CNOT2* KEGG pathway enrichment. **(B)** *SNW1* KEGG pathway enrichment.

Supplementary Table 1. Function of candidate reference genes.

SYMBOL	GENENAME	FUNCTION
RBM45	RNA binding motif protein 45	binding to poly(C) RNA, neural development
NRF1	nuclear respiratory factor 1	link the transcriptional modulation of key metabolic genes to cellular growth and development
BRAP	BRCA1 associated protein	negatively regulates MAP kinase activation by limiting the formation of Raf/MEK complexes
WDR33	WD repeat domain 33	cell cycle progression, signal transduction, apoptosis, and gene regulation
CNOT2	CCR4-NOT transcription complex subunit 2	be involved in mRNA synthesis, degradation, slicing, transport and localization
TIAL1	TIA1 cytotoxic granule associated RNA binding protein like 1	possesses nuleolytic activity against cytotoxic lymphocyte target cells
CIAO1	cytosolic iron-sulfur assembly component 1	Assist iron-sulfur protein assembly, modulate the transactivation activity of WT1
TARDBP	TAR DNA binding protein	regulate transcription and splicing, involved in the regulation of CFTR splicing
ZNF207	zinc finger protein 207	enable microtubule binding activity
HNRNPK	heterogeneous nuclear ribonucleoprotein K	influence pre-mRNA processing and other aspects of mRNA metabolism and transport
UBC	ubiquitin C	act as a polyubiquitin precursor
ACTB	actin beta	Protein involved in various types of cell motility
PUM1	pumilio RNA binding family member 1	serve as a translational regulator of specific mRNAs by binding to their 3' untranslated region
RPN1	ribophorin I	essential subunit of the N-oligosaccharyl transferase (OST) complex
RPL13A	Ribosomal protein LI3a	Structural component of the large 60S ribosomal subunit
PPIA	peptidylprolyl isomerase A	Accelerates the folding of proteins, catalyzes the cis-tans
PGK1	Phosphoglycerate kinase-1	Enzyme involved in glycolysis
IPO8	Importin 8	protein transport-nuclear import of proteins with a classical nuclear localization signal
HNRNPL	Heterogeneous Nuclear Ribonucleoprotein L	stably associated with hnRNP complexes, participate in processing of mRNA
SNW1	SNW domain-containing 1	signal transduction, regulation of transcription and splicing
CNOT4	CCR4-NOT transcription complex subunit 4	global transcriptional regulation, deadenylase, signal transduction, E3 ubiquitin ligase
